# A Collaborative Case Report Utilizing ChatGPT AI Technology of Traumatic Right Coronary Artery Dissection Resulting in Inferior Wall ST-Elevation Myocardial Infarction

**DOI:** 10.7759/cureus.35894

**Published:** 2023-03-08

**Authors:** Bhuvi Raxwal, Prashant Baisla, Jayant Nath

**Affiliations:** 1 Medicine, Access Health Care Physicians, Zephyrhills, USA; 2 Information Technology, University of the Cumberlands, Williamsburg, USA; 3 Cardiology, Memorial Healthcare System, Pembroke Pines, USA

**Keywords:** mva (motor vehicle accident), coronary stenting, acute myocardial infarction, chest trauma, st-elevation myocardial infarction, traumatic coronary artery dissection, post traumatic complications, post trauma ecg, artificial intelligence, chatgpt

## Abstract

Traumatic Coronary Artery Dissection (TCAD) is a rare but potentially life-threatening condition that can occur as a result of blunt chest trauma. This case report presents the case of a 67-year-old male who presented to the hospital after a motor vehicle accident with chest pain and ECG showing an inferior wall ST-elevation myocardial infarction.

Further imaging revealed a nondisplaced fracture of the right side of C2 and a left 12th rib. Coronary angiography showed a dissection in the distal segment of the right coronary artery. The patient was given heparin and underwent coronary stenting of the right coronary artery. The patient was monitored in the intensive care unit for any post-traumatic complications and was managed conservatively for the cervical fracture. This case report highlights the importance of prompt diagnosis and comprehensive treatment in TCAD cases to improve outcomes and prevent further complications.

## Introduction

Traumatic Coronary artery dissection (TCAD) is a rare and serious condition that occurs when a tear develops in the wall of the coronary artery, causing blood to enter and separate the layers of the artery, resulting in severe chest pain, arrhythmias, myocardial infarction, and even death if left untreated [1]. Trauma, such as a car accident, a fall, or a direct blow to the chest, can cause transection of the vessel leading to a TCAD. Treatment includes anticoagulants, anti-arrhythmic medications, and revascularization procedures such as angioplasty or coronary artery bypass grafting, depending on the severity of the case. Prompt diagnosis and treatment are essential for achieving positive outcomes and minimizing complications.

This case report presents a case of coronary artery dissection post-trauma in a 67-year-old male patient and discusses current management strategies.

## Case presentation

  A 67-year-old male who was a restrained driver sustained injuries following a high-impact motor vehicle collision. Specifically, the patient's pickup truck was struck perpendicularly on the passenger side, leading to multiple rollovers. The patient presented with head trauma and was extracted from the vehicle by a bystander without experiencing any loss of consciousness. Additionally, the patient exhibited contusions to the abdominal and chest wall, believed to be associated with seat belt restraint use.

**Figure 1:**
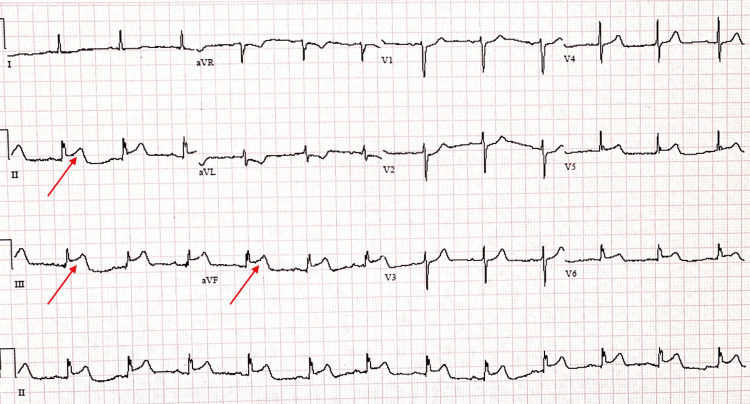
ECG. Demonstrate inferior wall ST elevation myocardial infarction (pointed by arrows).

The patient has a history of hypertension, diabetes, chronic kidney disease, and hypercholesterolemia. The patient complained of chest pain, and Electrocardiogram (ECG) showed inferior wall ST-elevation myocardial infarction (Figure 1). The patient reported no chest pain, syncope, or dizziness prior to the accident. 

Medical Services (EMS) transport was called to transfer the patient to a level 2 Trauma center, and additional imaging was obtained while awaiting transfer. The patient underwent a computed tomography (CT) scan of the cervical spine, chest, and abdomen and was transferred to a hospital with a level 2 Trauma center for further evaluation of C2 spine fracture and management of myocardial infarction. The patient was also noted to have a left 12th rib fracture. The patient's creatine was 1.5 mg/dl with a glomerular filtration rate (GFR) of 47, and troponin I was four on admission with peak troponin I of 12 ng/ml during the hospital stay.

Echocardiogram showed a normal ejection fraction with no valvular heart disease and no significant wall motion abnormalities. CT of the cervical spine showed a comminuted, nondisplaced fracture of the right side of C2 involving a portion of the right pedicle and a part of the right transverse foramen. The odontoid process was normal. CT of the chest demonstrated a left 12th rib nondisplaced fracture. Computed tomography angiography (CTA) of the chest, abdomen, and pelvis did not show aortic dissection or major organ injury. CT of the brain did not demonstrate any significant abnormalities.

**Figure 2:**
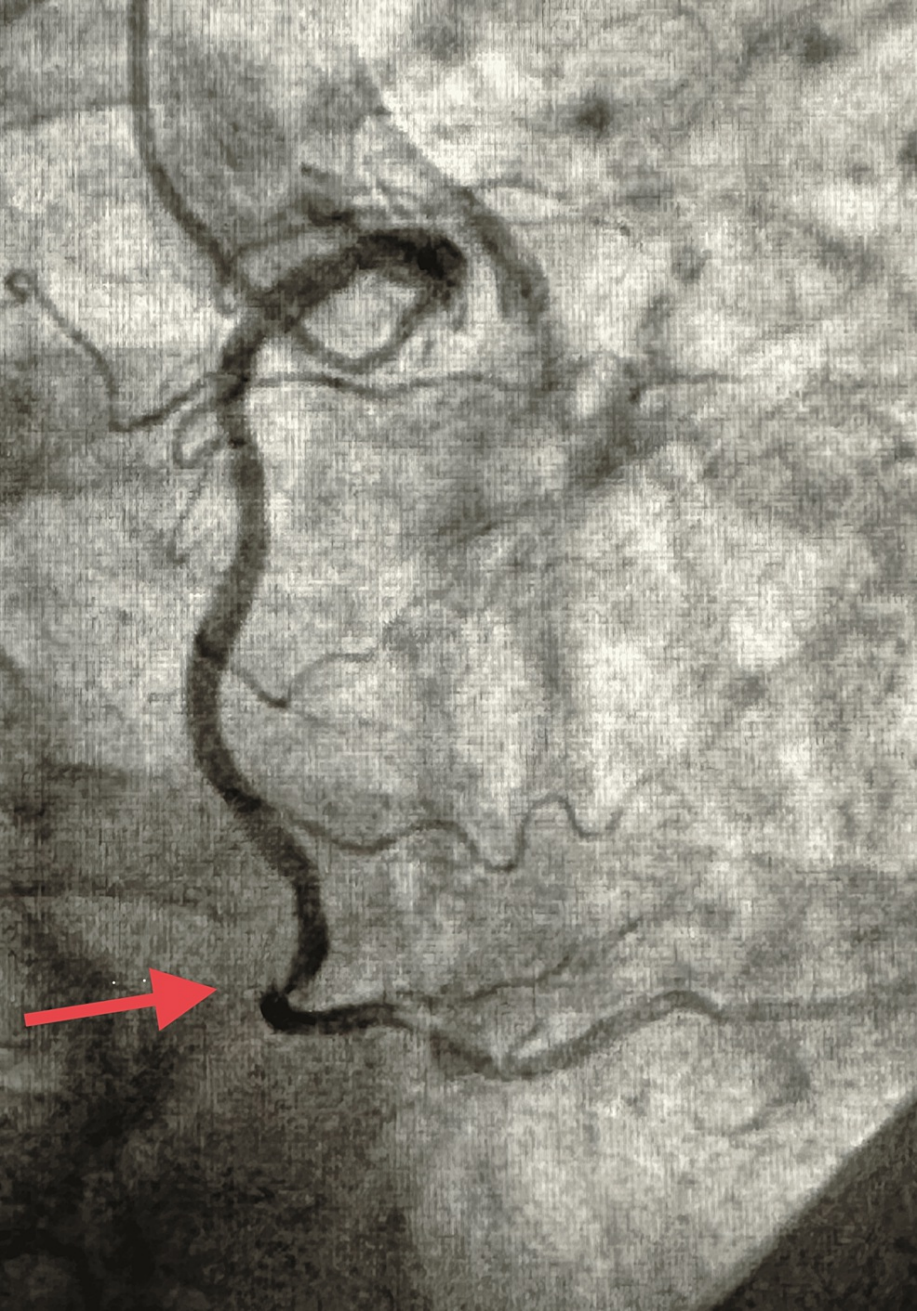
Distal Right Coronary dissection (pointed by arrow).

**Figure 3:**
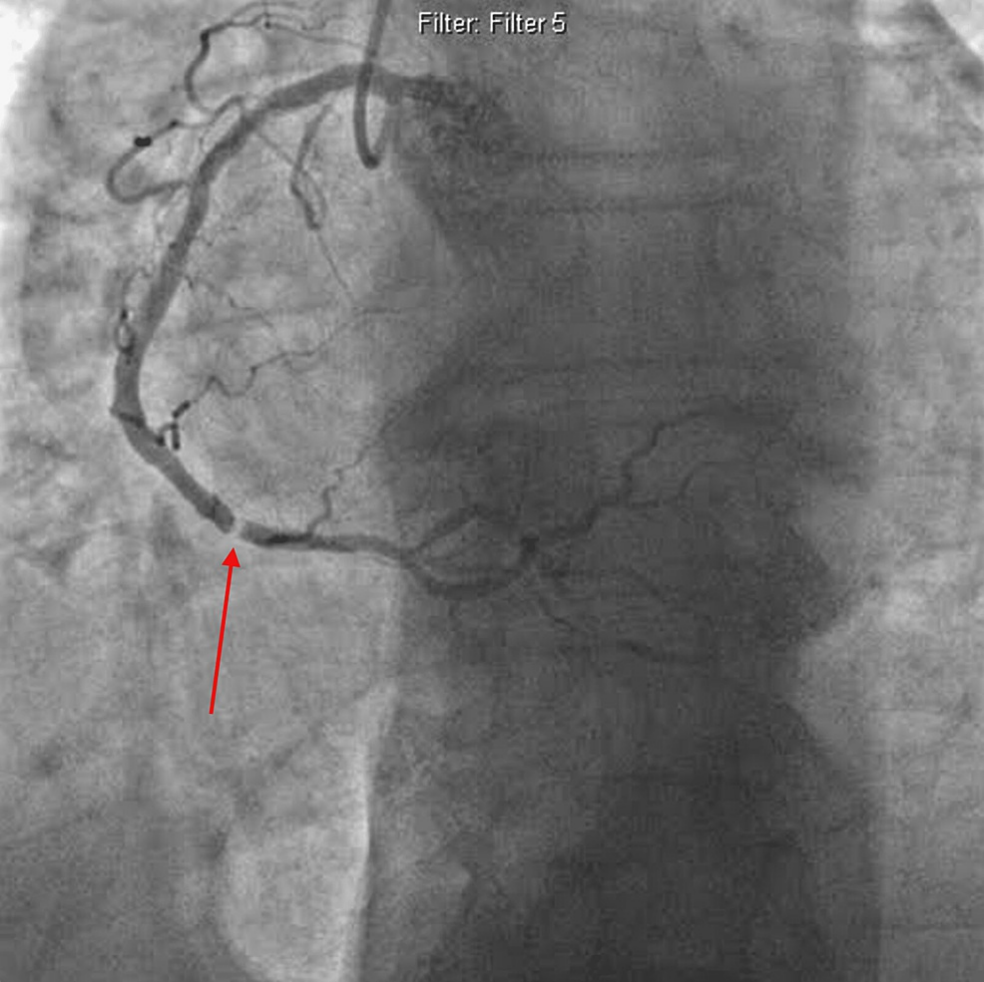
Distal right coronary dissection (pointed by arrow).

The patient was admitted to the hospital and underwent a thorough multidisciplinary team evaluation by trauma, CT surgery, and interventional cardiology in the emergency room. The decision was made to proceed with coronary angiography for further management of acute myocardial infarction. A coronary angiogram showed right coronary artery (RCA) dissection in the distal segment (Figures 2, 3).

**Figure 4:**
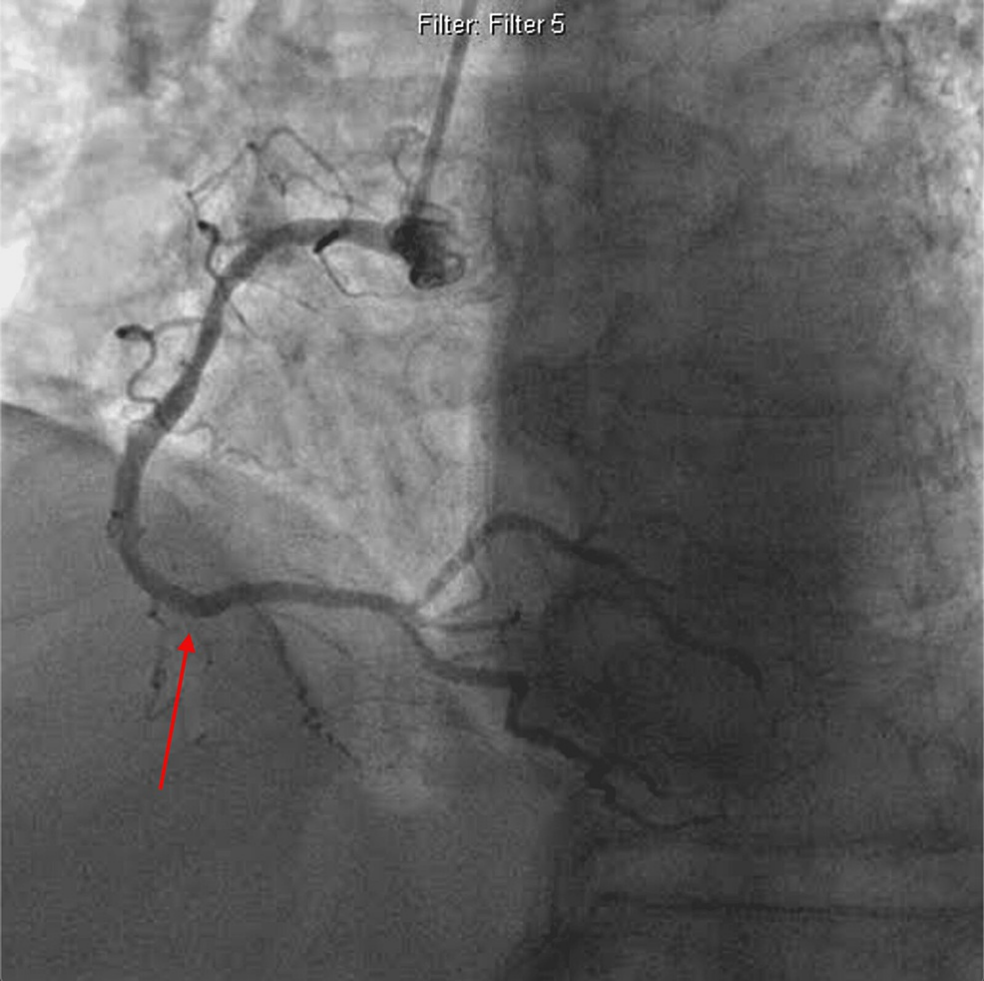
Distal Right coronary artery stenting (pointed by arrow).

The left anterior descending artery (LAD) is chronically occluded with collaterals; the left circumflex artery (LCX) with 50% stenosis; and normal ejection fraction (EF). The patient was given heparin, and because of ongoing chest pain and ST changes, it was decided to proceed with coronary intervention. Treatment included anticoagulation therapy, antiplatelet agents, and coronary stenting of the right coronary artery with a bare metal stent in view of possible anticipation of surgical intervention in the near future (Figure 4).

The patient was monitored in the intensive care unit for any possible cardiac arrhythmia and post-traumatic complications. During the hospital stay, the patient was evaluated by the neurosurgery team. Given the nondisplaced cervical fracture, the patient was managed conservatively with a cervical collar with close monitoring of symptoms for any neurological deterioration. Follow-up assessments were conducted to monitor the patient's progress and assess any complications. The results of the case report indicate that timely diagnosis and comprehensive treatment can improve outcomes in coronary artery dissection post-trauma cases.

## Discussion

Traumatic right coronary artery dissection is a rare form of injury caused by blunt trauma to the chest. The exact incidence of traumatic right coronary artery dissection is unknown; however, the incidence of cardiac-related complications in patients who presents to the ED with blunt chest trauma has been estimated to occur in approximately 0.1% of patients presenting with chest pain after blunt thoracic trauma or penetrating injuries involving the heart and great vessels [2]. The majority of cases are seen in young men due to their increased risk for physical trauma from activities such as sports and motor vehicle collisions [3]. However, the incidence may be underreported as TCAD can be difficult to diagnose and may be mistaken for other conditions, such as acute coronary syndrome. Chest pain related to the heart can be difficult to diagnose in trauma patients due to other injuries and tenderness in the chest area, especially in this patient who had suffered from a rib fracture.

Currently, there are no recommendations for routine ECG screening for all patients, regardless of the mechanism of their chest trauma. However, it's important to note that coronary artery dissection can happen to anyone, irrespective of their demographic, and can occur with any form of blunt thoracic trauma. Previous guidelines indicated that whether or not to get an ECG in cases of blunt chest trauma is at the discretion of the practitioner, which has been updated, and an admission ECG should be performed on all patients in whom blunt cardiac trauma is suspected and cardiac enzymes and transthoracic echo are also reasonable [4]. It is recommended to screen for cardiac injury even in cases of low-energy blunt trauma and even in young patients with a low risk of coronary atherosclerosis [5].

Traumatic coronary artery dissection presenting symptoms can be chest pain, shortness of breath, palpitations, dizziness/syncope, and arrhythmias [6]. Traumatic dissections are most commonly found in the LAD (76%) due to their vulnerable anatomic position in the heart [7]. The RCA (12%) and LCX (6%) are also affected, with the RCA being particularly vulnerable at its origin due to acceleration/deceleration injuries [8]. The exact mechanism for these dissections has yet to be fully understood.

This patient's history of trauma, clinical presentation, and diagnostic findings are consistent with the diagnosis of TCAD. The diagnosis of TCAD can be challenging and requires a high index of suspicion and a combination of clinical, laboratory, and imaging findings. The patient was treated with emergency stenting and had a successful outcome. However, it is important to note that TCAD can be associated with significant morbidity and mortality [9]. Given the potential for serious complications, it is important for healthcare providers to be aware of TCAD and to consider it in the differential diagnosis of patients with chest trauma.

Early diagnosis is essential for successful treatment, including revascularization with stenting or bypass surgery and medical management with anticoagulants and antiplatelet agents to prevent clot formation. Care must be taken when administering anticoagulation in trauma patients, as they may be at risk of bleeding due to other injuries. Close monitoring for recurrent dissection or complications is important to avoid long-term damage.

Use of ChatGPT

**Figure 5:**
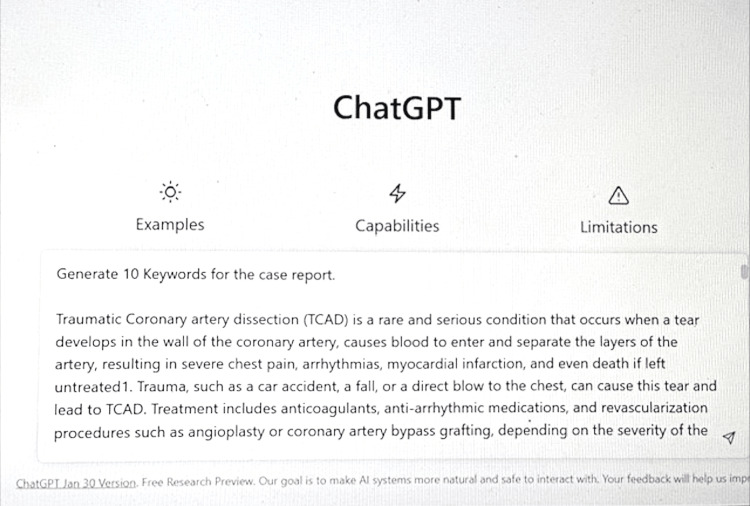
ChatGPT Query

The title, abstract, introduction, and conclusion sections were composed in part using the ChatGPT (Chat Generative Pre-trained Transformer) tool [10]. ChatGPTcan be a useful tool in the process of academic writing (Figure 5).

**Figure 6:**
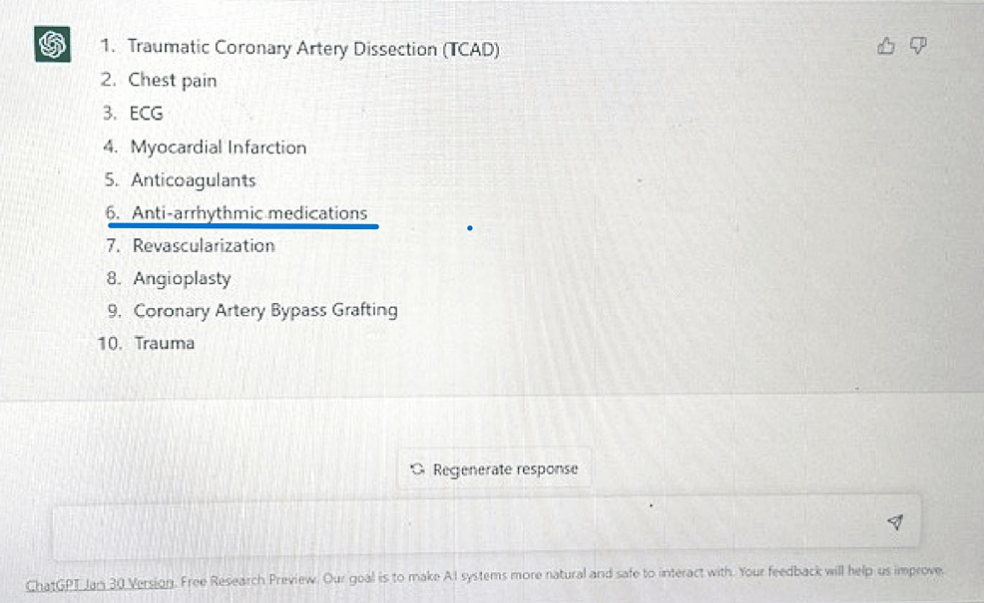
ChatGPT Query results. Highlighted irrelevant keyword.

However, it has certain limitations that should be considered when using it. Firstly, it lacks originality and may not be able to produce completely original ideas or arguments. Secondly, it lacks context and may not be able to make appropriate connections between different pieces of information (Figure 6); as exemplified in query results, the keyword anti-arrhythmic medications have no relevance in this case report. Thirdly, it has a limited understanding of citation and referencing and may not be able to properly cite references.

Additionally, it lacks critical thinking and is not able to critically evaluate research information. Lastly, it lacks creativity and is not able to come up with new ideas. Therefore, while ChatGPT can assist with generating text, it should not be used as a replacement for human writers in academic writing as it lacks the ability to understand the context and provide originality and critical evaluation of the topic.

In addition, we did explore other software, the OpenAI Playground beta version, which is a powerful, AI-driven platform that offers superior results compared to ChatGPT when it comes to writing research papers [11]. Its highly advanced natural language processing and text generation features are capable of producing far more accurate and sophisticated results. Furthermore, it allows for greater flexibility and customization, so users can generate results that meet their individual research needs and preferences. OpenAI Playground also offers superior control over randomness and data diversity through its nuclear sampling capabilities.

## Conclusions

In conclusion, traumatic coronary artery dissection is a rare but potentially life-threatening condition that can occur as a result of blunt chest trauma. The diagnosis can be challenging and often requires a combination of clinical, laboratory, and imaging findings. Early diagnosis and aggressive treatment are essential for a favorable outcome and to prevent further complications.
